# Advances in the clinical application of orthotic devices for stroke and spinal cord injury since 2013

**DOI:** 10.3389/fneur.2023.1108320

**Published:** 2023-02-17

**Authors:** Yinxing Cui, Shihuan Cheng, Xiaowei Chen, Guoxing Xu, Ningyi Ma, He Li, Hong Zhang, Zhenlan Li

**Affiliations:** Rehabilitation Medicine Department, First Hospital of Jilin University, Changchun, China

**Keywords:** orthotics, 3D-printing, motor dysfunction, stroke, spinal cord injury

## Abstract

Stroke and spinal cord injury are common neurological disorders that can cause various dysfunctions. Motor dysfunction is a common dysfunction that easily leads to complications such as joint stiffness and muscle contracture and markedly impairs the daily living activities and long-term prognosis of patients. Orthotic devices can prevent or compensate for motor dysfunctions. Using orthotic devices early can help prevent and correct deformities and treat muscle and joint problems. An orthotic device is also an effective rehabilitation tool for improving motor function and compensatory abilities. In this study, we reviewed the epidemiological characteristics of stroke and spinal cord injury, provided the therapeutic effect and recent advances in the application of conventional and new types of orthotic devices used in stroke and spinal cord injury in different joints of the upper and lower limbs, identified the shortcomings with these orthotics, and suggested directions for future research.

## 1. Introduction

Stroke and spinal cord injury (SCI) are common neurological disorders that can cause neurological dysfunctions (1, 2). Motor dysfunction is a common complication often accompanied by low muscle strength, muscular hypertonia, and limited joint activities. Serious complications, such as joint stiffness and muscle contracture, can easily occur if left untreated, significantly impacting the activities of daily living (ADL) and the long-term prognosis of patients (3).

Orthotic devices are special or general products developed using rehabilitation engineering technology that can prevent or compensate for the dysfunction in motor activities caused by neurological disorders. Orthotic devices can effectively reduce or overcome motor dysfunction and support rehabilitation training to improve movement and participation (4). Early use of orthotic devices with rehabilitation skills can rectify limb deformities and avoid secondary damage. The ADL and self-care ability of patients can be improved by improving their motor function and compensatory ability. Functional improvement may ease the burden on family and society and shorten rehabilitation (5, 6).

## 2. Methods

The first part briefly summarizes the epidemiological characteristics of stroke and SCI and shows the necessity for orthosis. The second section reviews progress with the clinical application of orthotic devices in stroke and SCI. In the second section, a literature search was conducted in November 2022, based on a selective search in the PubMed/MEDLINE databases to search the literature from January 1, 2013, up to September 30, 2022. We used search terms related to “stroke”, “spinal cord injury”, “orthosis”, “orthoses”, “orthotics”, “orthotic device”, “brace”, “splint”, and “arm sling”. The literature search was limited to articles published in English in which the full text was available. This manuscript mainly included prospective and retrospective research articles of upper or lower limb orthotic devices for patients with stroke and SCI. Studies that involved spinal orthoses, devices implanted in the body, orthoses with electrical/electronic components (or involving electrical stimulation devices), robotic devices, and orthoses unrelated to limb joints were not included. Studies not related to the improvement of motor function or ADL were also excluded. Fifty seven articles were selected to be included in this study.

## 3. Discussion

### 3.1. Epidemiological characteristics of stroke and spinal cord injury and the need for orthotic devices

Stroke is a common cause of hemiplegia. It is a group of acute cerebrovascular diseases that can induce many complications, including motor and cognitive dysfunction, aphasia/dysarthria, and psychological problems, which affect survivors' social activities and quality of life (7, 8). Motor dysfunction was the most common complication associated with stroke (9). It often has manifestations, such as low muscle strength, dystonia, and limited joint activities, which seriously affect the patient's balance, walking ability, and ADL (8). Stroke is characterized by a high prevalence in disability, recurrence, and mortality and is the second leading cause of death worldwide (10). In the United States, ~795,000 people experience a new or recurrent stroke each year. Approximately 7.0 million people over 20 years of age have experienced a stroke. The overall prevalence of stroke was ~2.5%. It is estimated that by 2030, there will be an increase of 3.4 million people with stroke in people over 18 years, and the prevalence will increase by 20.5% compared to 2012(11).

SCI is also a common central nervous system injury caused by traffic crashes, falls, and violence. SCI usually results in severe disruption of sensorimotor and autonomic nerve functions and may lead to severe physical and psychological problems in survivors. Tetraplegia and paraplegia are the most common sequelae of SCI (12), indicating that motor dysfunction occurs in the injury plane and is accompanied by abnormal muscle tension and pathological reflexes. Survivors may face permanent impairments, and only a few have completed neurological recovery. This can impose a heavy burden on individuals, families, and society. SCI can lead to severe morbidity and mortality and is estimated to affect 250,000–500,000 people annually (13). In Western Europe, the incidence of new cases of SCI is ~16–19.4 per million people annually (14).

In these neurological disorders, if spasticity, joint range of motion, and motor dysfunction are not reduced and corrected early, complications such as limited joint movement and stiffness, and muscle contracture will occur that can affect patients' quality of life. It is estimated that the global population of people with disabilities may exceed one billion, and more than half of them live in low- and middle-income countries (15). Although assistive devices may improve the function of people with disabilities, only 5–15% of people in need currently have access to assistive devices (16). Orthotics and prosthetics are important assistive devices. The orthotic device is an external application device used to restore and maintain anatomical and functional position and to assist the functions of the human body (17, 18). Common orthoses include upper limb orthosis, lower limb orthosis, and spinal orthosis according to the part of the body it is used. In addition, the main function of compression/containment orthosis is to improve limb stability by stabilizing the joints, and functional orthosis can control limb activities by stabilizing, supporting, strengthening, and protecting limbs based on joint stabilization and can also correct deformities and relieve pain (17, 19). Studies indicate that orthotics can effectively improve patients' function and prognoses and should be widely popularized (4–6).

### 3.2. Advances in the use of common orthotic devices

**Figure 1:**
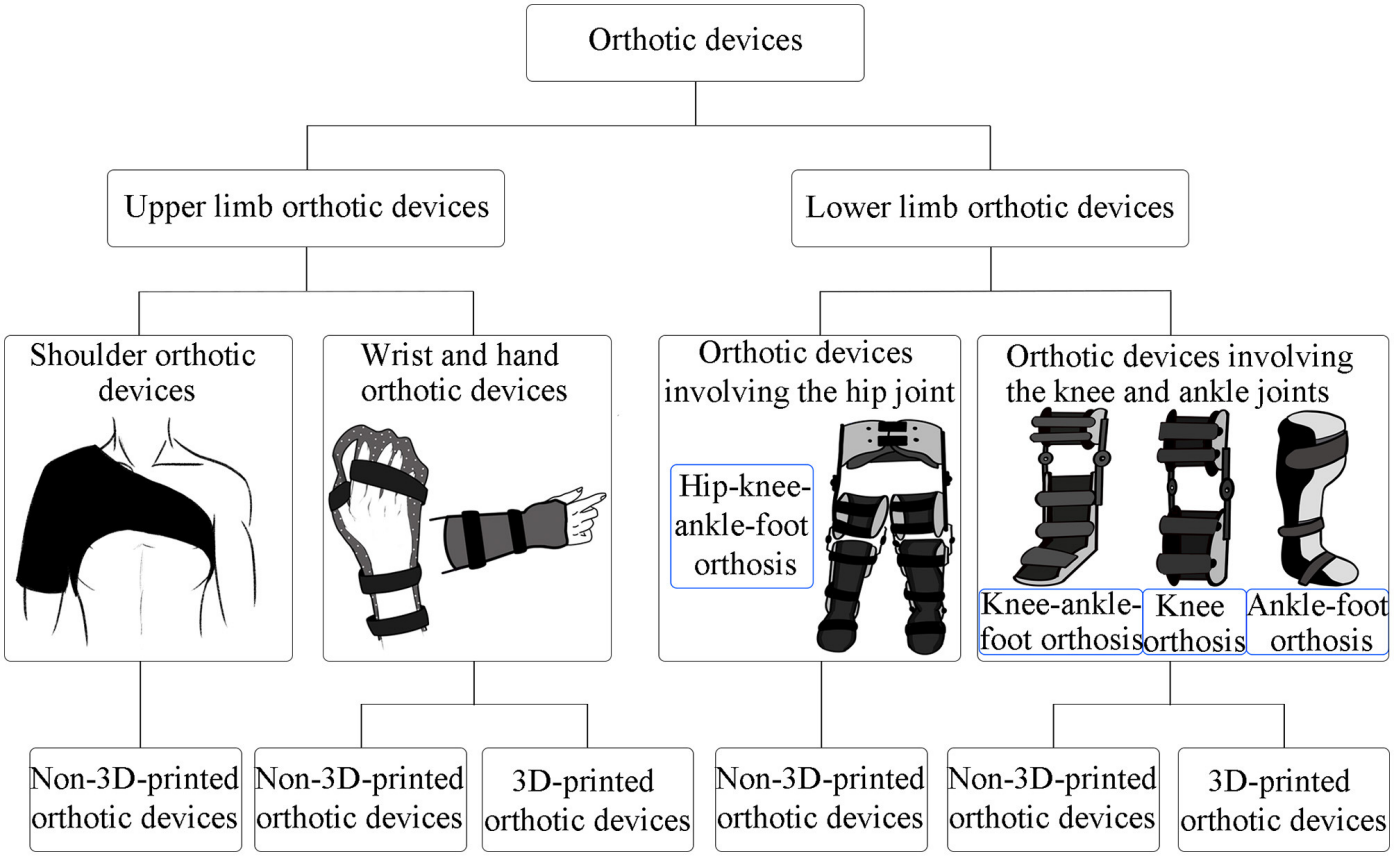
Classification of orthotic devices in the article.

To prevent contracture, limbs with motor dysfunction must maintain a joint range of motion. Methods to maintain the joint range of motion of limbs include normal limb position, stretching and standing training, and the use of orthotic devices. Early use of orthotic devices can play a role in early prevention, improve therapeutic effect, lay a stable foundation for later rehabilitation, and prevent joint deformities. It also helps control muscle tension, improve joint range of motion, prevent muscle contracture, and maintain physical alignment of the limbs. The classification of orthotic devices reviewed in this article is shown in Figure 1. The characteristics of the articles are summarized in Table 1.

**Table 1:** Characteristics of included studies.

**Limbs**	**Joints**	**Reference**	**Participants**	**Type of orthoses**	**Applied orthoses**	**Major findings**
Upper limb	Shoulder	Ada et al. (21)	(*n =* 46) Stroke	Non-3D-printed	Triangular sling, hemi-sling	Modified lap-tray combined with triangular sling showed no significant difference compared to hemi-sling in preventing GHS.
		Van et al. (22)	(*n =* 28) Stroke	Non-3D-printed	Shoulderlift, Actimove^®^ sling	Actimove^®^ sling: more pain at rest (*P =* 0.036). No sling: decrease in subluxation (-37.59% or 3.30mm).
		Hesse et al. (23)	(*n =* 40) Stroke	Non-3D-printed	New shoulder orthosis	Using orthosis significantly decreased the vertical distance between acromion point and the central point of the humeral head by an average of 0.8cm.
		Kim et al. (24)	(*n =* 41) Stroke	Non-3D-printed	Elastic dynamic sling, Bobath sling	Horizontal distance significantly decreased in the elastic dynamic sling group compared with the Bobath sling group (*P =* 0.006). No significant difference in motor and ADL functions between the groups.
		Jeong et al. (25)	(*n =* 57) Stroke	Non-3D-printed	Arm sling	Using arm sling could reduce the energy cost compared to no sling (*P <* 0.05), and walking distance of 6MWT was significantly increased (*P <* 0.05) among the patients with single cane and arm sling.
		Jeong et al. (26)	(*n =* 57) Stroke	Non-3D-printed	Arm sling	Using arm sling could reduce energy consumption and increase walking endurance compared with no sling (*P <* 0.01).
	Wrist and hand	Khallaf et al. (27)	(*n =* 24) Stroke	Non-3D-printed	Wrist-finger extension splint	Task-specific training and wrist-finger extension splint were effective in improving the results of nine holes peg test, FMA-UE, and joint range of motion (*P* ≤ 0.05).
		Wong et al. (28)	(*n =* 30) Stroke	Non-3D-printed	Dynamic hand orthosis	No significant difference in motor function improvement between task-oriented training combined with dynamic hand orthosis group and task-oriented training alone group.
		Lannin et al. (29)	(*n =* 9) Stroke	Non-3D-printed	SaeboFlex	Although using SaeboFlex showed no significant difference on the assessment scales, the hand function had a greater improvement trend than that of the usual rehabilitation group.
		Woo Y et al. (30)	(*n =* 5) Stroke	Non-3D-printed	SaeboFlex	Using SaeboFlex showed significant improvement in FMA-UE (*P <* 0.05).
		Zheng et al. (31)	(*n =* 40) Stroke	3D-printed, non-3D-printed	3D-printed orthosis, low-temperature thermoplastic plate orthosis	3D-printed orthoses significantly improved the modified Ashworth scale (*P =* 0.02), passive extension of wrist joint(*P <* 0.001), and FMA (*P <* 0.001) compared to low-temperature thermoplastic plate orthoses.
		Chen et al. (32)	(*n =* 6) Stroke	3D-printed	3D-printed multifunctional hand device	3D-printed multifunctional hand device significantly improved the ARAT scores, grip force and lateral pinch force (*P <* 0.05).
		Yang et al. (33)	(*n =* 8) Stroke	3D-printed	dynamic splint	Dynamic splints could improve the hand function and decrease the spasticity (*P <* 0.05).
		Wang et al. (34)	(*n =* 13) Stroke	3D-printed	3D printing fingerboard	3D printing fingerboard could improve the hand function and reduce the muscle tension.
		Huang et al. (35)	(*n =* 10) Stroke	3D-printed	3D-DHD	3D-DHD could significantly improve the results of BBT and the palmar pinch force test (*P <* 0.05).
		Kang et al. (36)	(*n =* 24) SCI	Non-3D-printed	Wrist-driven flexor hinge orthosis	Wrist-driven flexor hinge orthosis could improve pinch force (*P <* 0.001).
		Frye et al. (37)	(*n =* 19) SCI	Non-3D-printed	Prefabricated/custom-made resting hand splint	The outcomes of GRASSP had no significant difference between prefabricated and custom-made resting hand splints.
		Portnova et al. (38)	(*n =* 3) SCI	3D-printed	3D-printed wrist-driven orthosis	3D-printed wrist-driven orthosis could reduce assembly time and the cost of materials, and improve hand function.
Lower limb	Hip-knee-ankle	Yang et al. (39)	(*n =* 12) SCI	Non-3D-printed	HESWO, IRGO	HESWO could increase walking distance and speed, and reduce energy consumption compared with RGO (*P <* 0.05).
		Arazpour et al. (40)	(*n =* 4) SCI	Non-3D-printed	ARGO	Compared with the standard ARGO, the ARGO with a rocker sole could significantly improve walking speed, step length, hip flexion and extension (*P <* 0.05).
		Bani et al. (41)	(*n =* 4) SCI	Non-3D-printed	ARGO	ARGO with dorsiflexion assist AFO could significantly improve walking speed and stride length compared to that with SAFO (*P <* 0.05).
		Arazpour et al. (42)	(*n =* 5) SCI	Non-3D-printed	ARGO	ARGO with dorsiflexion assist AFO significantly improved walking speed and endurance compared to that with SAFO (*P <* 0.05).
		Samadian et al. (43)	(*n =* 6) SCI	Non-3D-printed	IRGO	Walking speed when using IRGO significantly improved after 4, 8, and 12 weeks compared to baseline (*P* = 0.010, *P* = 0.003, and *P* = 0.005).
		Arazpour et al. (44)	(*n =* 9) SCI	Non-3D-printed	IRGO	IRGOs with a reciprocating link significantly improved gait speed and step length compared to IRGO without it (*P <* 0.05).
		Karimi et al. (45)	(*n =* 5) SCI	Non-3D-printed	RGO, KAFO	Newly developed RGO significantly improved standing stability compared to KAFO (*P <* 0.05).
		Karimi et al. (46)	(*n =* 3) SCI	Non-3D-printed	RGO, KAFO	Compared with the KAFO, energy consumption of the newly developed RGO was significantly reduced (*P <* 0.05).
	Knee	Portnoy et al. (47)	(*n =* 31) Stroke	Non-3D-printed	Hinged soft KO	Using KO could significantly improve the results of BBS, 6MWT, 10MWT and TUGT (*P <* 0.05).
	Knee-ankle	Sato et al. (48)	(*n =* 112) Stroke	Non-3D-printed	KAFO	Using KAFO early could improve FIM gain (*P =* 0.032) compared with delayed using group.
		Maeshima et al. (49)	(*n =* 50) Stroke	Non-3D-printed	APS KAFO, traditional KAFO	APS KAFO was more suitable for patients with better motor function, traditional KAFO was more suitable for patients with severe symptoms.
		Talu et al. (50)	(*n =* 20) Stroke	Non-3D-printed	KIB, FLO	Among different combinations, KIB combined with FLO was the most helpful in improving the standing balance (*P <* 0.05).
	Ankle	Carse et al. (51)	(*n =* 8) Stroke	Non-3D-printed	SAFO	SAFO could significantly improve walking velocity, step length, and cadence of patients (*P <* 0.05).
		Pongpipatpaiboon et al. (52)	(*n =* 24) Stroke	Non-3D-printed	Thermoplastic AFO, APS AFO	AFO increased toe clearance (*P =* 0.038) and limb shortening (*P <* 0.0001), and diminished hip elevation due to pelvic obliquity (*P =* 0.003). No statistical difference between different AFOs.
		Tsuchiyama et al. (53)	(*n =* 32) Stroke	Non-3D-printed	Thermoplastic AFO, APS AFO	AFO could significantly improve gait stability (*P <* 0.05). However, in patients with mild ankle impairment, the results showed a worsening trend after wearing an AFO.
		Lan et al. (54)	(*n =* 20) Stroke	Non-3D-pringted	Plastic AFO	AFO could significantly improve walking capacity (*P <* 0.05).
		Do et al. (55)	(*n =* 17) Stroke	Non-3D-printed	Hybrid AFO, plastic AFO	Using AFO significantly increased walking speed compared to barefoot (*P <* 0.05). The hybrid and plastic AFOs showed similar effects in motor function.
		Rao et al. (56)	(*n =* 23) Stroke	Non-3D-printed	Plastic AFO	With AFOs, the results of the functional reach test significantly improved compared to those without orthoses (*P <* 0.05).
		Momosaki et al. (57)	(*n =* 1863) Stroke	Non-3D-printed	AFO	AFO significantly improved the FIM compared to no orthotics (*P <* 0.05).
		Zollo et al. (58)	(*n =* 10) Stroke	Non-3D-printed	SAFO, dynamic AFO	No significant difference between patients with solid and dynamic AFOs.
		Kim et al. (59)	(*n =* 9) Stroke	Non-3D-printed	Elastic band-type AFO, plastic AFO	The maximum dorsiflexion value of ankle joint increased significantly after using elastic band-type AFO (*P <* 0.005).
		Kim et al. (60)	(*n =* 10) Stroke	Non-3D-printed	Elastic AFO, plastic AFO	Postural stability index significantly improved with AFOs compared to no orthotics (*P <* 0.05). Elastic AFO improved some aspects of postural stability more substantially than hard plastic AFO.
		Farmani et al. (61)	(*n =* 18) Stroke	Non-3D-printed	Rocker bar AFO, SAFO	Rocker bar AFO significantly increased step length and gait velocity, and reduced the preswing time compared to SAFO (*P <* 0.05).
		Karakkattil et al. (62)	(*n =* 20) Stroke	Non-3D-printed	Double-adjustable AFO, PLS AFO	No significant difference between using double-adjustable AFO and PLS AFO in distance of 6MWT, gait symmetry and velocity.
		Nikamp et al. (4)	(*n =* 33) Stroke	Non-3D-printed	Rigid/semi-rigid/flexible non-articulated AFO	Using AFO could significantly improve the BBS, 6MWT, functional ambulation categories and TUGT in both groups (early or delayed provision) (*P <* 0.05). Early AFO provision could significantly improve the results of BBS and the Barthel index compared with delayed provision (*P <* 0.05).
		Nikamp et al. (63)	(*n =* 33) Stroke	Non-3D-printed	Rigid/semi-rigid/flexible non-articulated AFO	Early or delayed AFO provision did not show any difference on outcome measures after 26 weeks.
		Nikamp et al. (64)	(*n =* 26) Stroke	Non-3D-printed	Rigid/semi-rigid/flexible non-articulated AFO	Early or delayed AFO provision showed no kinematic differences for joint angles.
		Nikamp et al. (65)	(*n =* 20) Stroke	Non-3D-printed	Rigid/semi-rigid/flexible non-articulated AFO	Early or delayed AFO provision did not affect outcome measures.
		Nikamp et al. (66)	(*n =* 33) Stroke	Non-3D-printed	Rigid/semi-rigid/flexible non-articulated AFO	Early AFO provision increased the incidence of falls compared with delayed provision (*P =* 0.039), but 63.6% of falls occurred while the patient was not wearing an AFO.
		Pomeroy et al. (67)	(*n =* 105) Stroke	Non-3D-printed	AFO	The therapist-made AFO did not improve the effectiveness of conventional physical therapy.
		Pourhoseingholi et al. (68)	(*n =* 15) Stroke	Non-3D-printed	Spring damper AFO, PLS AFO	Newly developed spring damper AFO could significantly improve the results of the BBS, TUGT and ABC compared to the PLS AFO (*P <* 0.05).
		Yamamoto et al. (69)	(*n =* 36) Stroke	Non-3D-printed	AFO-OD, nonarticulated AFO	AFO-OD group showed more obvious improvement in ankle joint kinematics and kinetics than those of the nonarticulated AFO group.
		Kimura et al. (70)	(*n =* 8) Stroke	Non-3D-printed	AFO-OD	AFO-OD significantly improved gait parameters compared to without orthosis (*P <* 0.05).
		Yamamoto et al. (71)	(*n =* 40) Stroke	Non-3D-printed	AFO with plantar flexion stop, AFO-OD	After using orthotics, AFO-OD group had decreased thoracic tilt. But the AFO with plantar flexion stop group had increased pelvic forward tilt compared with no orthotics.
		Koller et al. (72)	(*n =* 10) Stroke	Non-3D-printed	Passive-dynamic AFO, hinged AFO	With the passive-dynamic AFO, improvements in gait-related parameters were observed in some participants.
		Tyson et al. (73)	(*n =* 139) Stroke	Non-3D-printed	Prefabricated PLS AFO, custom-made AFO	The user satisfaction and walking function had no significant difference between prefabricated and custom-made AFOs.
		Liu et al. (74)	(*n =* 12) Stroke	3D-printed	AFO	Compared with no AFO, gait velocity and stride length with AFO increased significantly (*P =* 0.001, *P =* 0.002). Although with no significant difference, the double limb support phase decreased with AFO.
		Hsu et al. (75)	(*n =* 7) Stroke	3D-printed, non-3D-printed	Anterior AFO, 3D-printed ideal training AFO	Ideal training AFO increased ankle dorsiflexion during the swing phase and extended the duration of paralyzed lower limb standing phase compared with conventional AFO.
		Arazpour et al. (76)	(*n =* 5) SCI	Non-3D-printed	SAFO, hinged AFO	Step length: barefoot 26.3 ± 16.37cm, SAFO: 31.3 ± 17.27cm, hinged AFO 28.5 ± 15.86cm. Only step length between SAFO and barefoot showed significant difference (*P <* 0.05).

APS, adjustable posterior strut; 6MWT, 6-minute walk test; FMA, Fugl-Meyer assessment; FMA-UE, FMA of upper extremity; BBT, box and blocks test; FIM, functional independence measure; BBS, Berg balance scale; 10MWT, 10-m walk test; TUGT, timed up and go test; ABC, activities-specific balance confidence; ARAT, action research arm test; GRASSP, graded redefined assessment of strength, sensation and prehension; GHS, glenohumeral subluxation; SCI, spinal cord injury; ADL, activities of daily living; 3D, three-dimensional; 3D-DHD, 3D printed dynamic hand device; HESWO, hip energy storage walking orthosis; RGO, reciprocating-gait orthosis; ARGO, advanced RGO; IRGO, isocentric RGO; KAFO, knee-ankle-foot orthosis; KO, knee orthosis; KIB, knee immobilization brace; FLO, Foot Lifter Orthosis^®^ AFO, ankle-foot orthosis; AFO-OD, AFO with oil damper; PLS AFO, posterior leaf spring AFO; SAFO, solid AFO.

#### 3.2.1. Upper limb orthotic devices

Hemiplegia due to stroke and quadriplegia due to SCI can cause upper limb motor dysfunction. Upper-limb orthotic devices are widely used in stroke and SCI. They can prevent and correct upper limb deformities, keep the limbs in functional position, provide traction to prevent joint contracture, partially compensate for the function of disabled muscles, and help to treat upper limb motor dysfunction.

##### 3.2.1.1. Shoulder orthotic devices

Shoulder orthotic devices are commonly used to treat glenohumeral subluxation (GHS). GHS, also known as shoulder subluxation, is a common complication in hemiplegia. GHS can cause loss of range of motion due to the instability of the shoulder joint. Approximately 80% of stroke patients with hemiplegia may experience GHS (20), and if left untreated may cause shoulder pain, upper extremity edema, and limited shoulder joint movement. A study compared the efficacy of hemi-sling to a lap-tray combined with a triangle sling in GHS among acute stroke survivors. The result showed no significant difference between the two groups in preventing subluxation, pain, contracture, or movement limitation (21). This suggests that further studies may be needed to find effective shoulder support devices for patients with GHS. Van et al. (22) also compared different arm slings and found that the shoulderlift that directly supports the shoulder joint was more efficient than the Actimove^®^ sling in reducing pain. However, subluxation was reduced only in the control group without slings, suggesting that orthoses may affect active correction. Although studies have shown that wearing orthoses is helpful for recovery in GHS, they should be removed promptly if necessary. The selection of orthotics and the appropriate time for wearing them may require further research.

X-ray findings suggest an improvement in GHS in some studies (23, 24). In a study, radiography revealed that wearing an orthosis reduced the vertical displacement of the glenohumeral joint in stroke patients (23). In another study using an elastic dynamic shoulder sling in stroke patients with GHS, radiography showed that the horizontal distance from the humeral head to the glenoid fossa improved compared to the control group (Bobath sling) (24). Considering that orthoses provide immediate improvement of GHS, and different orthoses have different effects on GHS recovery, it is necessary to adapt the best orthotic devices. Meanwhile, the results showed that the improvement in motor function was more pronounced after 8 weeks than after 4 weeks in the group (24). This suggests that the length of wearing time affects the functional improvement. However, the results also indicated no significant difference in motor and ADL functions between the groups (24). Studies suggest that improving motor function is an important method to recover from GHS, and further studies may be needed to consider suitable orthoses and GHS improvement methods for patients.

Interestingly, wearing shoulder orthotics also affected gait efficiency. Some studies have shown that patients with GHS after stroke wore shoulder support arm slings, which could reduce energy consumption and increase walking distance (25, 26). This suggests that posture correction may improve motor function. Dysfunction of different parts may affect each other, and rehabilitation after stroke should be comprehensive.

##### 3.2.1.2. Wrist and hand orthotic devices

Depending on the disorders, the wrist and hand orthotic devices can take various forms, such as wrist stabilization, wrist-hand stabilization, and wrist-finger stabilization. The biomechanical principle is to assist in the extension of the wrist and finger joints (77). Hemiplegic spasm is a common complication; the incidence of hemiplegic spasm in the 1st year after stroke is between 33 and 78%, and the incidence of contracture is at least 50% (78). Early prevention and treatment, such as passive stretching, can increase muscle extensibility and effectively reduce muscle spasms to improve the recovery of upper limb function. Wrist and hand orthotic devices assist in the stabilization of the wrist and hand in a functional position and may be considered an effective method of passive stretching to reduce wrist flexor spasticity. Wrist and hand orthotic devices often prevent wrist and finger contractures in hemiplegic survivors, but their effectiveness is unclear (79). A study using task-specific training combined with wrist-finger extension splints in hemiplegic patients, showed effective improvements in finger dexterity, upper limb motor function, and range of motion of the wrist and hand joints (27). However, another study suggested that task-oriented training combined with dynamic hand orthosis did not significantly improve motor function compared to task-oriented training alone in patients with subacute stroke (28). Further studies are needed to determine the timing and circumstances of wearing orthoses, considering that not all cases using orthotic devices had beneficial effects on motor function improvement compared with no orthotics. However, sample size and other factors may have influenced the results.

Patients with cervical SCI are prone to quadriplegia, and after rehabilitation treatment, the recovery of motor function is often incomplete, and orthotic assistance is needed. The wrist-driven flexor hinge orthosis, a device designed to restore hand function by providing three-point prehension, has been used in patients with SCI and has shown a significant increase in pinch force (36). Using orthoses can improve the patient's hand function, which is helpful for ADL, such as eating. A study comparing prefabricated and custom-made resting hand splints among SCI patients showed no statistical difference (37). Although custom-made orthotic devices are generally recommended in clinical practice, sometimes their advantages are minimal, and they have the disadvantages of being time-consuming and expensive. In some cases, prefabricated orthotics can also be used. However, the custom-made orthotic devices require further improvement.

There are some special orthoses for the recovery of motor function in patients. One study showed that SaeboFlex, a spring-assisted orthosis, helped improve hand dexterity in patients with almost complete loss of hand function after stroke (29). Additionally, a study showed that SaeboFlex significantly improved upper limb motor function in patients with stroke (30). Considering that if static hand orthoses cannot effectively improve distal upper-limb motor function, it is necessary to use appropriate orthoses to improve hand function effectively.

Currently, conventional wrist and hand orthotic devices have certain disadvantages. Some of them are bulky, and their customization is time-consuming. With technological advances, numerous new orthotic devices have emerged, including custom-made three-dimensional (3D) printed orthoses. With 3D printing technology, orthoses can be accurately designed using computer graphics program, which can solve the problems of time-consuming manufacturing and difficult customization of conventional orthotic devices. The materials used for 3D printing are also readily available (80), and 3D-printed orthoses can be made of lightweight, ventilating, and biodegradable materials (81). A study compared two different types of wrist-hand orthoses, and the results showed that the therapeutic effect of 3D-printed orthoses was better than that of low-temperature thermoplastic plate orthoses. Compared with the other orthosis, 3D-printed orthosis could better reduce the spasticity of stroke patients and had an important effect on improving the motor function of the wrist joint (31). Since 3D-printed orthoses can be customized more accurately through software and are more adaptable to patients than conventional orthoses, they may provide better support. Some studies have shown that 3D-printed orthotic devices can effectively improve patients' hand function (32–34) and compared the effects of wearing time (3 weeks vs. 3 months). The results showed that the grip strength and hand function of stroke patients tended to improve with an increase in wearing time, although the difference was insignificant (34). Considering that 3D-printed orthotics can effectively improve patients' hand function, prolonging wearing time will not cause adverse reactions but will further improve the motor function of the patients. Furthermore, a 3D-printed dynamic hand device (3D-DHD) was used to supplement task-oriented training in stroke survivors. The results showed that the improvement in hand function in the 3D-DHD group was greater than that in the task-oriented training alone group (35). This suggests that 3D-printed orthoses combined with appropriate rehabilitation methods can more effectively improve the motor function of patients.

3D-printed orthoses can also be used in patients with SCI. A study has shown that using 3D printing technology to make wrist-driven orthoses could reduce hands-on assembly time and the cost of the material. In addition, hand function in patients with SCI could improve (38). Considering with 3D printing technology, we developed an orthosis that can accurately adapt to a user, and its function is not inferior to that of conventional technology. Although 3D printing technology may require more conditions, it is worth promoting and can compensate for the many defects of conventional orthoses.

#### 3.2.2. Lower limb orthotic devices

Hemiplegia and SCI-induced tetraplegia/paraplegia are common causes of lower limb motor dysfunction. Lower-limb orthotic devices can support body weight, prevent and correct lower-limb deformities, effectively compensate for the function of paralyzed muscles, and limit unnecessary activities of the lower-limb joints. They can improve posture while standing and walking and help treat lower limb motor dysfunction. Moreover, lower-limb orthotic devices may help improve patients' ADL (82).

##### 3.2.2.1. Orthotic devices involving the hip joint

The hip-knee-ankle-foot orthosis (HKAFO) is the most common hip joint orthotic device according to the literature search results and is the main hip joint orthotic device reviewed in this paper. The HKAFO was used to stabilize the hip, knee, and ankle joints. It is suitable for patients with extensive lower limb muscle paralysis and assists patients in standing and walking. A reciprocating gait orthosis (RGO) is a type of HKAFO. Different types of HKAFOs have different effects on lower limb motor function. A study comparing the newly designed hip energy storage walking orthosis (HESWO) and RGO, suggested that SCI patients wearing HESWO had more significant gait improvement and lower energy consumption than those wearing RGO, considering that HESWO can provide a more energy-efficient gait (39). Arazpour et al. added a rocker sole to advanced RGO (ARGO) and found improvements in walking function compared to ARGO with a flat sole among patients with SCI (40). Two studies compared two kinds of ARGOs; and the results suggested that ARGO with dorsiflexion-assisted ankle-foot orthosis (AFO) was better than that with solid AFO in improving gait function in patients with SCI (41, 42). These studies suggest that the influence of different orthotic components on motor function improvement should be considered. Through continuous research with orthotic components, appropriate orthotic devices should be adapted according to the functional status of the patients.

One study showed that using isocentric RGO (IRGO) in patients with SCI could significantly improve walking capacity (43). Another study compared IRGO with and without the reciprocating link, and the results showed that the reciprocating link was useful in improving the walking ability of patients (44). IRGO is effective in improving walking parameters in patients with SCI. However, the sample sizes of these studies were small. To determine which orthotic devices are suitable for users, we need to increase the sample size for further studies to identify appropriate orthoses in clinical practice.

Two studies investigated whether controlling the hip joint improves motor function. They compared the standing stability between RGO and a knee-ankle-foot orthosis (KAFO) (45, 46). The results showed that compared with the KAFO group, patients with SCI wearing the newly developed RGO were more stable in standing at rest and performing tasks, especially when standing at rest (45). Meanwhile, the energy cost decreased significantly and walking style improved (46). Considering that hip joint control is helpful for standing stability, for paraplegic patients with SCI, orthotics with hip control may help improve motor function.

Although one study showed no significant difference in gait speed between powered gait orthosis and IRGO (83), orthotics with electrical/electronic components have been widely used in recent years to improve walking capacity in patients with SCI (84). However, this paper focused on orthotics without electrical/electronic components. Therefore, these orthotic devices were not reviewed in detail.

##### 3.2.2.2. Orthotic devices involving the knee and ankle joints

KAFO and AFO are the most common orthotic devices involving the knee and ankle joints. The KAFO is used in hemiplegic patients with unstable knee and ankle joints and lumbar paraplegia. It can support, stabilize, and limit the movement of the joints and is suitable for knee and ankle joints rehabilitation. Of course, there is also a knee orthosis (KO) for simple knee joint stabilization (47). AFO is widely used for foot and ankle deformities, such as strephenopodia, strephexopodia, and foot drop.

KAFO is widely used to stabilize lower limb segments during walking. However, only a few paraplegic patients discharged from the hospital continue to use KAFO. KAFO gait requires upper limb muscle strength, increases gait fatigue and may lead to upper limb musculoskeletal injury. Consequently, the KAFO is often used for standing posture or gait training rather than functional gait (85). However, some studies have shown that KAFO may positively affect patient recovery. A previous study showed that using a KAFO early could significantly improve the ADL in stroke patients (48). Another study showed that in hemiplegic patients, the adjustable posterior strut KAFO was more suitable for patients with better motor function, whereas traditional KAFO was suitable for patients with severe symptoms and difficulty obtaining practical walking ability (49). It is beneficial for patients to wear orthoses early, and different orthoses are suitable for patients with different functional statuses.

One study used three different applications: knee immobilization brace (KIB), KIB combined with Foot Lifter Orthosis^®^ (FLO), and KIB combined with rigid taping, suggesting that KIB combined with FLO was the most helpful strategy for improving the balance of hemiplegic patients (50). Considering that simultaneous control of the knee and ankle joints is helpful for the balance of hemiplegic patients, the effect of FLO on ankle joint stabilization is better than that of rigid taping. Therefore, the KAFO, which covers both the knee and ankle joints, is most widely used for patients who need to stabilize the knee joint. However, an orthotic device that stabilizes the knee joint can also positively improve motor function. Moreover, a study using hinged soft KO among stroke patients showed that KO prevented knee hyperextension, significantly improved balance and walking distance, and reduced walking time (47). Patients can improve their walking ability by controlling the knee joint and preventing knee hyperextension.

Some studies conducted gait analysis for stroke patients with and without AFO, and the results indicated that AFO effectively improved walking ability, gait stability (51–55), balance (56), and might improve ADL (57). Furthermore, AFO reduces compensatory strategies during walking (52). However, in some patients with mild ankle impairment, the results showed a worsening trend after wearing an AFO (53). Stabilizing the ankle joint can stabilize the lower limb, effectively regulate the posture, and help improve walking capacity. However, different effects may occur depending on the severity of the patient's condition, which requires further study.

Foot drop is a common complication in hemiplegic patients. One study showed that the solid and dynamic AFO had no significant difference in controlling foot drop (58). Some studies have compared elastic AFO and hard plastic AFO with no AFO. The results showed that compared with patients without orthoses, those with orthotic devices had improved motor function (59, 60). Furthermore, elastic band-type AFO could improve foot drop better than hard plastic AFO (59), and postural stability tended to improve (60). Research has shown that plastic materials may limit the ankle joint, resulting in insufficient ankle dorsiflexion (59). Foot drop may require ankle joint stabilization; however, a stiffer material might not provide the best support. Soft materials can also provide good ankle joint stabilization and improve user comfort.

Some studies have compared the different types of AFOs. A study comparing solid AFO with hinged AFO during treadmill training in patients with SCI showed that solid AFO could improve step length compared to hinged AFO, although with no statistical difference (76). Another study suggested that a rocker bar AFO might improve walking capacity better than a solid AFO (61). Considering that different orthotic devices have different effects on patient function, further studies are needed to adapt the best orthosis under different conditions. Moreover, Do et al. showed that wearing a hybrid AFO was similar to a plastic AFO in motor function, but the hybrid AFO was lighter and more satisfactory (55). Another study compared a double-adjustable AFO with a posterior leaf spring AFO (PLS AFO) and found no significant differences in walking capacity (62). The results suggest that the selection of orthoses requires many considerations. When there is no significant difference in functional improvement, the appropriate orthosis should be selected according to factors such as wearing comfort and patient satisfaction.

Some studies have compared the duration of use of orthotic devices. A study comparing the early provision of AFOs with delayed provision showed that both groups had significant improvement in walking function after wearing AFO, and the improvement of balance was more pronounced in the early provision group (4). This suggests that using an AFO early significantly affects the recovery of lower limb motor function. However, the effectiveness of early AFO use in patients with stroke paralysis with foot drops is controversial. Some studies have shown that early or delayed AFO provision after stroke did not affect outcomes (63–65), However, providing AFO had a positive short-term effect on ankle kinematics in the early phase after stroke (65). In addition, another study showed that using an AFO early increased the risk of falls in hemiplegic patients, but it was important to note that 63.6% of falls occurred while the patient was not wearing an AFO (66). Considering that patients who have adapted to AFO gait may be more prone to falls when they do not wear orthoses, attention should be paid to the use of orthoses when motor function has not sufficiently improved. Notwithstanding, wearing an AFO is still necessary for stroke patients, and the most appropriate time to wear orthoses may require further study. A study suggested that using quick-made AFOs by therapists did not improve the effectiveness of conventional physical therapy (67). However, quick-made orthotics are an option for patients to have custom-made orthotics at an early stage of the disease (67). While the effectiveness of early or delayed wearing of orthotics remains controversial, further exploration and improvement with orthotic devices are needed.

Some AFOs have dampers. A study has shown that the newly developed AFO with spring damper is superior to the PLS AFO in improving balance (68). Studies of AFO with an oil damper (AFO-OD) have suggested that AFO-OD might significantly improve ankle joint motor function and gait parameters in stroke patients (69, 70). Another study conducted a gait analysis after rehabilitation with different AFOs. The results showed that the AFO-OD group had decreased thoracic tilt, but the AFO with plantar flexion stop group had increased pelvic forward tilt (71) after wearing orthotics. Adding dampers may optimize the function of AFO and improve motor function. Meanwhile, AFO-OD can better avoid dislocation of thorax and pelvis when walking, and can guide a more stable and natural gait.

With the development of orthotics, custom-made orthotics have become increasingly common and sophisticated. In a study that personalized the passive-dynamic AFO, improvements in parameters related to walking function were observed in some participants (72). However, another study showed that compared with prefabricated orthotics, customized orthotic devices showed no improvement in walking function and user satisfaction (73). Considering that custom-made orthoses may improve patients function from a new perspective through different components, they need further research and are actively promoted in clinical practice.

3D printing technology can also be used to fabricate lower limb orthotic devices. A study showed that after stroke patients wore 3D-printed AFO, their gait speed and stride length improved, and the double limb support phase decreased (74). Motion feedback can also be used for orthotics. A study suggested that a 3D-printed AFO with motion feedback in stroke patients improved walking function better than conventional AFO (75). This suggests that 3D-printed orthoses exhibit good performance and are comparable to conventional orthoses. 3D printing technology has potential benefits in design and production and can be actively promoted.

## 4. Conclusion

In this article, we reviewed conventional and new types of orthotic devices for stroke and SCI according to the different joints of the upper and lower limbs. Conventional orthotic devices are widely used and can effectively improve motor function. Custom-made orthoses are generally recommended; however, sometimes, there are no significant differences in efficacy or user preference between prefabricated and customized orthotic devices. In addition, custom-made conventional orthotic devices are sometimes time-consuming. Nowadays, new orthotics and various components are constantly being developed, which tend to be durable, lightweight, ventilating, and intelligent, and the kinematics of these devices are very close to the anatomy of the human limb, sometimes even in the form of human-computer interactions. However, some devices are still in development stages and cannot be widely and immediately used in clinical practice. The direction of future research on orthotic devices is to improve the functions of conventional orthotic devices and develop new types of devices. Further research is needed to make them more consistent with clinical practice, help patients improve motor function, rebuild their confidence, and enable them to return to their families and society faster.

## Author contributions

YC drafted the manuscript. SC and XC performed the literature search and extracted the articles. GX, NM, HL, and HZ assisted with drafting and revising the manuscript. ZL conceived and designed the manuscript. All authors contributed to the article and approved the submitted version.
